# Breeding Selection for U.S. Siberian Huskies Has Altered Genes Regulating Metabolism, Endurance, Development, Body Conformation, Immune Function, and Behavior

**DOI:** 10.3390/genes16111355

**Published:** 2025-11-10

**Authors:** Heather J. Huson, Krishnamoorthy Srikanth, Karolynn M. Ellis

**Affiliations:** 1 College of Agriculture and Life Science, Cornell University, Ithaca, NY 14853, USA; ks786@cornell.edu; 2 College of Veterinary Medicine, Cornell University, Ithaca, NY 14853, USA; kme59@cornell.edu

**Keywords:** signatures of selection, F_ST_, runs of homozygosity, ROH, arctic breed

## Abstract

**Background:** The Siberian Husky has evolved as a versatile dog capable of traversing over 1600 km in extreme Arctic conditions, being a competitive show dog in the American Kennel Club, or a favorite pet for companionship. Modern genomics provides an opportunity to explore the biological implications of selection within the Siberian Husky breed for the purpose of sledding, show, or pet. **Methods:** We identified regions of genetic selection associated with sledding, show, or pet purposes using a whole-genome panel of 234 K SNPs from 237 Siberian Huskies. We assessed allelic variation using Wright’s F_ST_ and selective sweeps with runs of homozygosity (ROH). **Results:** Genomic and morphometric measurement principal component analyses identified population structure aligning with breeding purpose. In total, 118 SNPs demonstrated significant allelic variation (F_ST_ ≥ 0.6) and 22,598 ROH segments were identified within the Siberian Husky breed. ROH islands (*n* = 91) highlighted selective sweeps, whereas homozygosity association tests characterized regions of the genome under differential selection between populations. Genes within regions were assessed using GO and KEGG pathway analysis for biological insight. Pet dogs showed selection for olfactory performance genes, whereas show dogs were selected for immune function, tissue and nervous system development, and cytoskeletal motor activity. Sledding Siberian Huskies were selected for the development of muscle organs, lung vasculature, limbs, bones, eye structure, and pigmentation, plus genes influencing lipid metabolism and glucose transport. **Conclusions:** In all, this provides the first evidence of the biological impact of genetic selection within a breed for the distinct sledding, show, and pet purposes while simultaneously maintaining overall population uniformity to meet breed standards.

## 1. Introduction

The Siberian Husky was developed in Alaska in the early 1900s from Arctic dogs imported from the Chukchi, Koryak, Yukagir, and Kamchadal people, indigenous peoples from the northeastern-most part of Siberia [1,2]. Prior research comparing the genome of modern (2000s) and historic (1925) Siberian Huskies to DNA from Arctic dog fossils predicted at least two Arctic dog lineages existing at the end of the Pleistocene, around 12,800 mya, one of which leads to the modern Siberian Husky [3]. These early Siberian Huskies were known for their thick, double coats, medium-size frame, and exceptional endurance and hardiness in extreme Arctic conditions when working as sled dogs. They were smaller in stature compared to other Arctic sled dog contemporaries and became renowned for their speed and endurance in early sled dog racing competitions such as the All Alaska Sweepstakes [1].

The Siberian Husky became a recognized dog breed registered by the American Kennel Club (AKC) in the United States in 1930 [4]. This was followed by the establishment of the Siberian Husky Club of America (SHCA) in 1938 as an organization dedicated to promoting the selective breeding of the Siberian Husky to meet breed standards and preserve its natural qualities, educate people about the breed, and conduct AKC-sanctioned matches and specialty shows [2]. Siberian Husky breed standards for body weight range from 20 to 27 kg for males and 16 to 23 kg for females. Males should be 53–60 cm in height at the withers, whereas females range from 51 to 56 cm [4].

In 2024, the Siberian Husky breed was ranked 24th of 202 AKC breeds for popularity based on annual breed registration statistics [4]. With over a century of breeding, the Siberian Husky continues to be a popular sled dog with teams competing in extreme endurance races such as the Iditarod where they traverse 1688 km across harsh Alaskan terrain or sprint “short” distances of 5–35 km [5,6]. The Siberian Husky breed has also gained historic recognition with a win as Best in Show at the renowned Westminster Kennel Club Show in 1980 [4]. As pets, Siberian Huskies are known to be affectionate to people and good with other dogs, very playful and energetic, and relatively adaptable, but shed seasonally and can be willful when training [4].

Underlying the versatility of the Siberian Husky is the selective breeding within the population for these different purposes of sledding, showing, and as pets. As a closed breeding population, according to the rules of breed registration and the necessity to adhere to breed-specific characteristics in appearance and general behavior, Siberian Huskies have established their own unique genetic signature distinguishing them from other dog breeds [7,8]. However, research has also shown that there is genetic population structure within the breed that aligns with their selection for pet, sledding, or show purposes [3]. Anecdotally, observations have also noted variation in body conformation distinguishing purpose groups. To this end, this study aimed to identify divergent selection within the Siberian Husky breed associated with breeding purpose as a sledding, show, or pet dog. Selection was identified across the genome using Wright’s fixation index (F_ST_) and runs of homozygosity (ROH) mapping on a panel of 200 K single-nucleotide polymorphisms (SNPs). In addition, 18 morphometric measurements were assessed to determine the extent of morphological variation within the breed associated with breeding selection. Importantly, gene ontology and pathway enrichment analysis were performed using genes identified in regions of selection to understand the biological implications of within-breed selection for varying purposes.

## 2. Materials and Methods

### 2.1. Sample Collection and Genotyping

This study included 237 Siberian Huskies, all of which were registered with the American Kennel Club (AKC). Of these, 165 dogs were directly sampled for DNA, while genotypes for a further 72 Siberian Huskies were obtained from owners. Sex information was collected for all the samples, while 18 morphometric measurements [9] were collected for the 165 dogs that were directly sampled. A single set of morphological measurements was collected by one of three trained assistants on each dog using a flexible plastic measuring tape with dogs standing on a flat surface and following procedures described in Sutter et al., 2008 [9] (Supplemental File S1). Differences among groups for each morphometric trait were assessed using one-way ANOVA after assessing data for normality using the Shapiro–Wilk test and homogeneity of variances using Levene’s test. When ANOVA testing was significant, Tukey’s Honestly Significant Difference (HSD) test was applied for pairwise comparisons (α = 0.05). Anecdotally, variation in body conformation has been noted, particularly between Siberian Huskies used in sledding versus showing. Morphometric measurements provided an opportunity to objectively evaluate this observation and compare results to genomic findings. Animal handling and sample collection were approved by the Cornell University Institutional Animal Care and Use Committee (protocol #2014-0121).

The dogs were grouped into five categories based on owner-reported usage and breeding purpose history: Pet (*n* = 20, 13 owners), Show (*n* = 32, 20 owners), Sled (Sprint [*n* = 84, 7 owners] and Distance [*n* = 44, 2 owners]), Pet–Sled (*n* = 15, 4 owners), and Show–Sled (*n* = 42, 16 owners). Pet dogs were those bred strictly as companion animals. They were not intended for showing or sledding and did not originate from breeding targeting use in the show or sled categories. Show dogs were those bred and used specifically for AKC-sanctioned breed shows or competitions. The Sled dogs were dogs bred strictly for sledding competition alone. Within the Sled category, Sled—Distance described dogs who had competed in distance races over 1000 km (i.e., Iditarod or Yukon Quest), whereas Sled—Sprint dogs competed in races shorted than 50 km that were recognized by the International Sled Dog Racing Association (ISDRA) [6]. Show–Sled dogs were defined as show dogs meeting the regulation for “the sled dog class at specialty shows” or “the working/showing trophy.” These dogs must have competed in ten ISDRA-sanctioned events on snow with a minimum of 4 miles per heat [2]. The Pet–Sled category included dogs bred as companion dogs, where the owners sledded with the dogs as a hobby. They did not compete in sanctioned races.

Either a buccal swab or whole blood was collected from 165 dogs. The buccal swabs were collected using Performagene^®^ PG-100 cheek swab (DNA Genotek, Stittsville, ON, Canada), and DNA was isolated following the manufacturer’s instructions. Whole-blood samples were collected from the cephalic vein in 3 mL EDTA tubes, and DNA was isolated using in-house buffers and a two-step lysis and salt-out method, as previously described [10]. The quality of the DNA was assessed using a spectrophotometer (Epoch microplate spectrophotometer, Santa Clara, CA, USA). The samples were genotyped on the EMBARK customized Illumina CanineHD BeadChip (Embark Veterinary Inc., Boston, MA, USA). Genotypic data for an additional 72 Siberian Huskies, genotyped on the same EMBARK array, were acquired from owners. The initial genotype data included a total of 234,990 SNPs. The genotypes were filtered for quality using PLINK v.1.9 [11,12]. Only autosomal SNPs were retained using the following thresholds: individual call rate was set to 0.95 (-mind 0.05), SNP call rate was set to 0.95 (-geno 0.95), minor allele frequency pruning was set to 1% (-maf 0.01), and SNPs exhibiting significant deviation from the Hardy–Weinberg Equilibrium (Observed Frequency/Expected Frequency < 0.05 or >1.1) were excluded to minimize potential genotyping errors and ensure data quality. Unmapped SNPs and SNPs on the sex chromosomes and from mitochondrial DNA were also excluded. Post quality control, 124,479 SNPs were retained for further analysis.

### 2.2. Principal Component Analysis (PCA)

The population structure within the Siberian Husky breed was assessed using PCA on the genotype data. PCA was performed using the GCTA v.1.91 software (–autosome–autosome-num 38–make-grm–pca 2) [13]. A scatterplot was generated to visualize the first and second principal components using ggplot2 [14] in R v.4.3.2 [15].

PCA was also performed on the 18 morphometric measurements capturing body height, length, and girth, as well as bone lengths and circumferences using the ‘prcomp()’ function in R v.4.3.2 [15]. Only the top 5 principal components (PCs) were assessed and the reported proportion of variance explained (PVE) was calculated based on these five PCs. The loadings of the first four principal components were visualized using ggplot2 [14]. We tested significant differences in PCA scores between groups using one-way ANOVA after testing for assumptions of normality and homogeneity.

### 2.3. Signatures of Selection—Marker-Based F_ST_

Marker-based F_ST_ was one method used to identify signatures of selection within the Siberian Husky population. Marker-based F_ST_ is a measure of population differentiation due to allelic variation between populations, with scores closer to 1 signifying a greater degree of differentiation and scores closer to 0 representing more similarity between populations [16]. The F_ST_ analysis was performed with VCFtools 0.1.17 [17]. Pairwise F_ST_ values were calculated to quantify genetic differentiation between all groups, as well as a one-vs-all approach to identify group-specific differentiation across the genome. F_ST_ values ≥ 0.6 were considered significant as this threshold represents a more stringent criterion than the commonly used top 0.1% of F_ST_ values reported in other studies. Significant markers were annotated to the nearest gene in the CanFam 3.1 [18] genome assembly.

### 2.4. Signatures of Selection—Runs of Homozygosity (ROH)

Regions demonstrating selective sweeps commonly due to positive genetic selection of desired traits were identified through ROH. ROH are continuous segments of DNA where an individual has inherited the exact same genetic sequence on both chromosomes. ROH were identified using a consecutive runs approach as described by [19], using the R package detectRUNS [20] with the following criteria: (1) a minimum window size of 55 SNPs, (2) a minimum physical length of 1000 kb was required for an ROH segment, (3) a maximum of 1 heterozygous call was permitted within a run, (4) a maximum gap of 1000 kb was allowed between consecutive SNPs, and (5) a maximum of one missing SNP was permitted within a run. The choice of parameters was based on the recommendations of Gorssen et al. [21].

The identified ROHs were further categorized into four length classes: 0 to <6 Mb, 6 to <12 Mb, 12 to <24 Mb, and ≥24 Mb. For each length category, the frequency and average length of ROHs were computed for the different populations. Genome-wide inbreeding based on ROHs (*F_ROH_*) was calculated as the proportion of the autosomal genome covered by ROHs, following the equation described by Purfield et al. [22].

$$F_{\left\{ROH\right\}}=\frac{\sum L_{\left\{ROH\right\}}}{L_{\left\{AUTO\right\}}}$$

where *F_ROH_* is the inbreeding coefficient, *L_ROH_* is the total length of all ROHs identified per animal, and *L_AUTO_* is the total length of the autosomes covered by SNPs. In this study, *L_AUTO_* was estimated to be 2,201,878 kb. Additionally, *F_ROH_* was also calculated for each of the four ROH length categories to assess the contribution of different ROH lengths to the overall inbreeding level. Differences among groups (pet, show, sled—sprint, and sled—distance) for each ROH length class and genome-wide *F_ROH_* were assessed using one-way analysis of variance (ANOVA) after confirming data normality and homogeneity of variance. When the ANOVA indicated a significant effect (*p* < 0.05), Tukey’s Honestly Significant Difference (HSD) post hoc test was applied to identify pairwise differences between groups. All analyses were performed in R v.4.3.2.

To identify ROH islands indicative of potential selective sweeps within specific Siberian Husky populations, the frequency of SNPs within ROH segments was calculated for each population. These frequencies were visualized as Manhattan plots using the CMplot package [23] in R v.4.3.2 [15]. A threshold of 50% was then applied to identify an ROH island, meaning that an SNP was considered part of an ROH island if it was located within an ROH in at least 50% of individuals in the population.

### 2.5. Genome-Wide Homozygosity Association Analysis

To identify significant associations of ROH between Siberian Husky populations, clusters of ROH were identified and used in a genome-wide homozygosity association test. Clusters of ROH common across the entire Siberian Husky population were identified using SVS software v8.9.1 (Golden Helix Inc., Bozeman, MT, USA), following the approach described by Huson et al. [24]. ROH segments were first detected using the following criteria: (1) a minimum run length of 1000 kb containing at least 55 SNPs, (2) allowing up to 1 heterozygous genotype and 1 missing genotype per run, (3) with no gaps >1000 kb between consecutive SNPs, (4) and a minimum SNP density of 1 SNP per 150 kb. A consensus ROH observed in 20 or more individuals was defined as an ROH cluster. These clusters represent genomic regions where the same homozygous SNPs are consistently found across individuals, suggesting selective pressure. The frequency of homozygous SNPs within each cluster was then calculated, and the first SNP was then used to represent the region in a genome-wide homozygosity association test, implemented in SVS, based on the method described by Lencz et al. [25]. This analysis aimed to identify genomic regions under differential selective pressure between Siberian Husky populations by comparing the presence of ROH clusters across groups. The underlying methodology is similar for identifying both ROH islands and clusters. However, downstream analyses were different and required differing input files. We chose to maintain consistency with other publications and separated these two analyses based on their downstream output.

### 2.6. Gene Annotation and Pathway Enrichment Analysis

Genes within ROH islands/clusters or within 1 MB of a marker showing greater than 0.6 F_ST_ differentiation were used for pathway enrichment analysis to ascertain the functional implications of genetic selection. Genes were annotated using biomart [26] R package, mapping against CanFam 3.1 genome assembly [18]. Functional annotation analysis was performed using the KEGG and GO databases. Subsequently, functional enrichment analysis was carried out using the gProfiler tool to identify significantly enriched pathways and GO terms (FDR < 0.05). The results were visualized with ggplot2 [14] R package. Redundant GO terms were summarized using REVIGO [27].

## 3. Results

### 3.1. Genetic Population Structure

**Figure 1:**
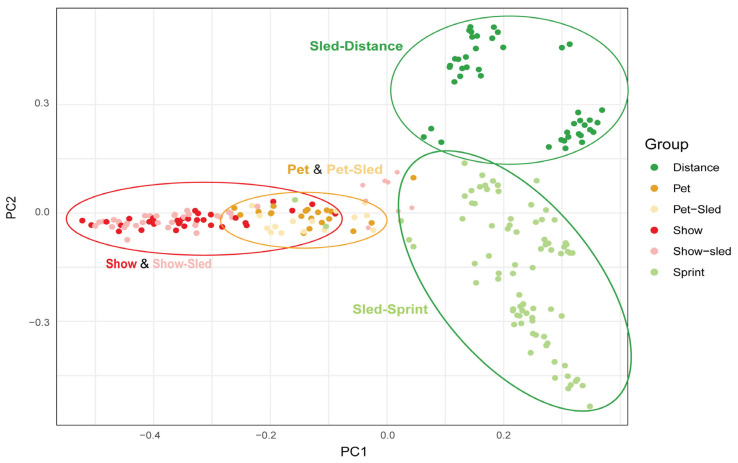
Genomic Principal Component Analysis based on Single-Nucleotide Polymorphism (SNP) Genotypes. This scatter plot shows the genetic relationships among individual Siberian Husky dogs based on their genotypes. Each point represents a single individual with its position determined by the first two principal components (PC1 and PC2) derived from genomic markers. Point colors reflect owner-reported usage and breeding selection as Show (red), Show—sled (pink), Pet (orange), Pet—sled (yellow), Sled—distance (dark green), or Sled—sprint (light green).

Genomic PCA identified the population structure within the Siberian Husky breed, reflecting owner-reported usage and selective breeding for sledding, pet, or show purposes (Figure 1). PC1 with an eigenvalue of 9.92, explaining 20.30% of the genetic variation, predominantly distinguished sledding from show dogs. Pet dogs were intermediate but overlapped the show and show–sled dog groups to some extent. PC2 with an eigenvalue of 9.21, representing 18.86% of the genetic variation, separated the two sub-categories of sled dogs—distance and sprint—from one another. The dogs from the two distance kennels sampled are also noticeably separated from one another (Figure 1—dark green high, left versus dark green slightly lower, right). The pet–sled dogs generally clustered with the pet dogs, and most of the show–sled dogs clustered with the show dogs, as expected based on the respective group’s pet or show breeding priority, respectively. However, there were two kennels of show–sled dogs that clustered more closely with sled—sprint dogs as opposed to show dogs, demonstrating a greater degree of variation in the breeding selection among show–sled dogs than the other dual-use dogs. Using genomic PCA results, all pet–sled dogs were combined with the pet dogs (*n* = 35, owners = 17), the show–sled dogs overlapping the show group were combined with the show dogs (*n* = 66, owners = 34), and the two kennels of show–sled dogs overlapping with sled dogs were combined with the sled—sprint group (*n* = 92, owners = 9). No change in group was made for dogs whose owners reported a single group regardless of genomic PCA results. In addition, for some analyses, the subcategories of sled—distance and sled—sprint were combined to represent all sled dogs (*n* = 136, owners = 11). This provided the categories of pet, show, and sled, as well as the subcategories of sled—distance and sled—sprint for further analyses.

### 3.2. Morphological Variation

**Figure 2:**
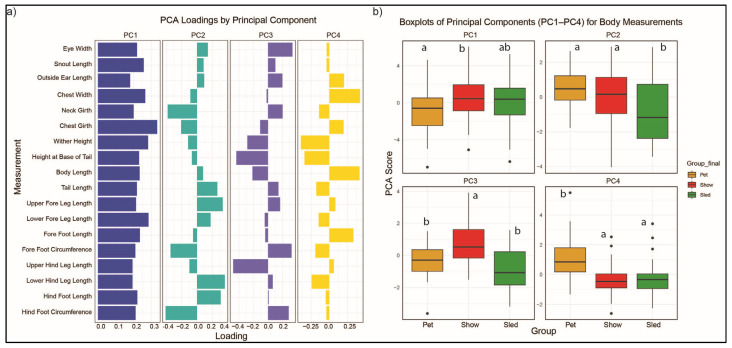
Principal Component Analysis of 18 Morphological Measurements. (**a**) This plot displays the loadings (correlations) of each morphological measurement onto the first four principal components (PC1—blue, PC2—cyan, PC3—purple, and PC4—yellow). Each bar represents a specific body measurement, and its length and direction indicate the strength and direction of its contribution to that principal component. Each panel illustrates group-level variation in morphometric traits. (**b**) Box plot of the pet (yellow), show (red), and sled (green) populations shows the distribution of PCA scores for each principal component (PC1, PC2, PC3, and PC4). Letters above the boxes indicate Tukey HSD groupings; groups that do not share a letter are significantly different (*p* < 0.05).

Eighteen morphometric measurements including heights, lengths, widths or girths, and bone circumferences were also assessed using PCA (Figure 2, Supplementary Figure S1). Together, the first four principal components described 62.06% of the genetic variation in body size. PC1 (EV = 5.47), individually accounting for 30.39% of the genetic variation, reflected overall body size, with all measurements positively correlated to one another. Significant differences in PCA scores were found between pet and show dogs in PC1. In general, pet dogs tended to have wider body measurements in girth and width, sled dogs were taller, and show dogs were consistently the smallest in width, height, and length.

PC2 (EV = 2.36; 13.10%) largely captured the morphological change in girth, foot circumference, and leg lengths, with the sled dog group differing significantly from the pet and show groups. Specifically, PC2 segregated pet, then show, then sled dogs with an inverse relationship of smaller neck and chest girth and smaller foot circumferences combined with longer tails, longer upper and lower foreleg, longer lower hind leg, and longer hind foot lengths. PC3 (EV = 1.85, 10.29%) distinguished dogs that were shorter in stature with wider faces, larger neck girth, and thicker bones from those that were longer and taller, particularly with longer hind leg length, and thinner in girth and bone circumference. The variation captured in PC3 segregated show dogs (*p*-value < 0.05), which had the highest scores, from the other two groups, with pet dogs being intermediate and sled dogs being the lowest. PC4 (EV = 1.49, 8.28%) distinguished pet dogs (*p*-value < 0.05) from both show and sled dogs, who were similar to one another. Variation largely distinguished dogs who were short, with a long body, and a wider chest.

In total, 11 of the 18 measurements showed significant differences between Siberian Husky pet, show, and sled populations (Table 1). There was no discernable difference in size variation between groups based on sex, with the pet cohort having a nearly equal representation of males (*n* = 16) and females (*n* = 19), and the show and sled groups having slightly more males (show *n* = 28; sled *n* = 86) than females (show *n* = 28; sled *n* = 50). Sled dogs had the most measurements that were significantly different from the other populations, which included shorter, lower hind leg length and upper foreleg length, taller heights at the withers and base of the tail, and narrower eye width. Show dogs were significantly different, with shorter upper hind leg length, whereas pet dogs had wider chests. Uniquely, body length was the only variable with significantly different averages between all three populations. Pet, then sled, then show dogs exhibited bodies from the longest to the shortest, with more than an inch separating each population average.

### 3.3. Marker-Based F_ST_

**Figure 3:**
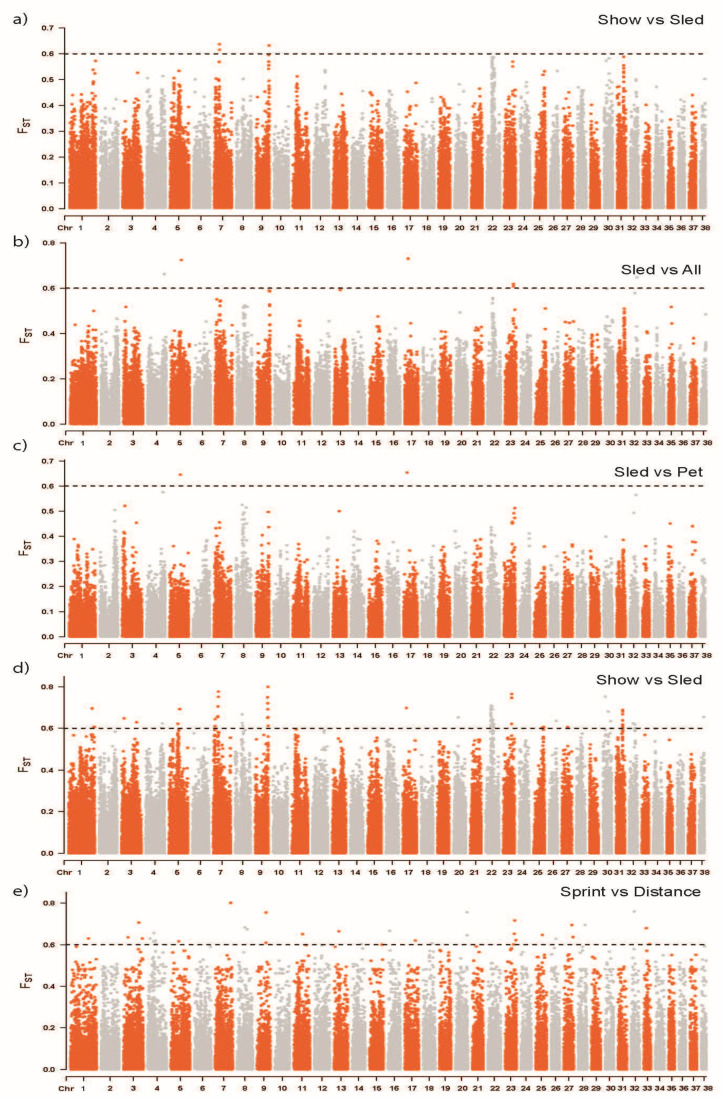
Manhattan plots of genetic differentiation (F_ST_) between Siberian Husky populations. The individual Manhattan plots display a genome-wide scan for regions of high genetic differentiation, as measured by F_ST_. Each plot shows a comparison between two groups, with plots (**a**,**b**) representing show or sled Siberian Huskies as compared to all other Siberian Huskies, respectively. Plots (**c**–**e**) show a pairwise comparison between two individual populations or the sled subpopulations of sprint and distance. No significant results were obtained for pet versus all other Siberian Huskies or pet versus show dogs (plots not shown). The *x*-axis represents the chromosomes (from 1 to 38), and the *y*-axis shows the F_ST_ value for each genomic marker. The dashed horizontal line represents a significance threshold of 0.6.

Marker-based F_ST_ identified regions of selection due to allelic frequency differences in pairwise comparisons across populations. Show, sled, and pet populations were individually compared against all remaining dogs (e.g., show versus pet and sled combined) to identify allelic variation distinct to these main population categories. Then, pairwise comparisons across each group and across the sled subcategories were conducted to further identify regions of differentiation. A cutoff of F_ST_ ≥ 0.6, representing substantial genetic differentiation between populations, identified 118 SNPs in the multiple pairwise comparisons (Figure 3, Supplementary Table S1). Specifically, two SNPs on CFA 7 and one SNP on CFA 9 (Figure 3a) differentiated show Siberian Huskies from all other Siberian Huskies. Individual SNPs on CFA 5, 17, and 32 differentiated sled Siberian Huskies from all others (Figure 3b). No markers of F_ST_ ≥ 0.6 differentiated the pet population, nor was there significant variation between the pet and show populations. However, there were two SNPs on CFA 5 and 17 that differentiated the pet and sled Siberian Huskies (Figure 3c). These were the same SNPs (CFA5.50239253 and CFA17.15791076) distinguishing sled from all others. The greatest degree of variation was between the show and sled populations with 79 SNPs spanning CFA 1, 3, 5, 7, 8, 9, 17, 20, 22, 23, 25, 27, 30, 31, 32, and 38 (Figure 3d). This comparison also had the SNP (CFA9.6027889) with the highest F_ST_ of 0.80. The other major source of allelic variation was between the distance and sprint sledding groups with 31 SNPs spanning CFA 1, 3, 4, 5, 8, 9, 13, 14, 15, 16, 17, 18, 20, 23, 26, 27, 28, 32, and 33 (Figure 3e).

### 3.4. Runs of Homozygosity

**Figure 4:**
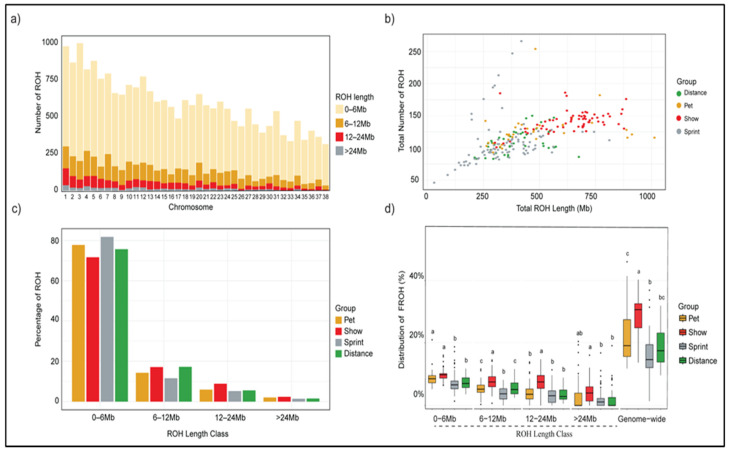
Summary of runs of homozygosity in Siberian Husky dogs. (**a**) The stacked bar chart shows the distribution of ROHs across chromosomes. The height of each bar represents the total number of ROHs on that chromosome. (**b**) The scatter plot shows the relationship between the sum of the length ROH and the total count of ROH across individuals. (**c**) The percentage distribution of ROH by length class for each Siberian husky population. (**d**) Inbreeding estimates (*F_ROH_* (%)) for different ROH length classes and genome wide for each group. The boxplots show the distribution of *F_ROH_* and letters above the boxes indicate statistically significant differences between groups with each ROH class.

ROH provide insight into the inbreeding level, timeframe of inbreeding occurrence, and identification of selective sweeps across the genome. A total of 22,598 ROH segments were detected. Figure 4 characterizes ROH based on their count, frequency, length, and contribution to inbreeding within Siberian Huskies and within the different groups of Siberian Huskies. ROH < 6 Mb in length dominate each chromosome by count, with progressively longer ROH (6.1–12 Mb, 12.1–24 Mb, and >24 Mb) becoming less frequent (Figure 4a). Show and pet dogs tend to have a longer combined total length of ROH due to more ROH being present in their genomes than in those of sled dogs (Figure 4b). A comparison of the percentage of varying ROH lengths present in the categories of pet, show, sled—sprint, and sled—distance dogs indicates that short ROH (<6 Mb) are higher in pet and sled dogs, whereas longer ROH (>6 Mb) are significantly higher in show dogs (Figure 4c). Across all ROH length classes, differences among groups were highly significant (ANOVA, *p* < 0.001), and Tukey’s post hoc tests identified show dogs as significantly different from all other groups for longer ROH length classes (>6 Mb) (Figure 4d).

Genome-wide inbreeding (*F_ROH_*) also varied among groups (Figure 4d). Show dogs exhibited the highest median *F_ROH_* (30.7%), followed by pet (19.1%) and sled—distance dogs (17.5%), while sled—sprint dogs had the lowest level (14.6%). Tukey’s post hoc comparisons confirmed that show dogs had significantly higher genome-wide *F_ROH_* than all other groups (*p* < 0.01) and that sled—sprint dogs differed significantly from pet dogs (*p* < 0.01) (Figure 4d).

**Figure 5:**
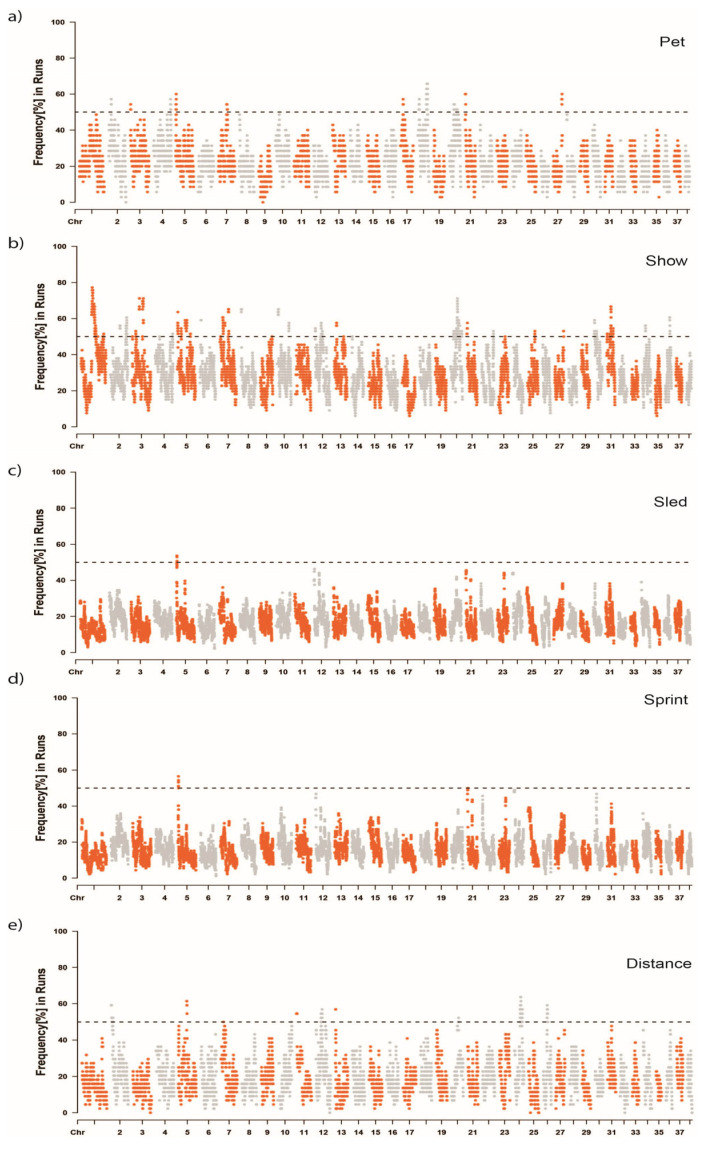
Frequency of SNPs in runs of homozygosity (ROH) across chromosomes for different Siberian Husky groups. The Manhattan plots show the frequency of SNPs in ROH in each Siberian Husky group ((**a**) pet, (**b**) show, (**c**) sled, (**d**) sprint, and (**e**) distance). The *x*-axis represents the chromosomes (from 1 to 38), and the *y*-axis shows the frequency percentage of each genomic marker being found within an ROH. The dashed horizontal line represents a significance threshold of 50%.

The frequency of individual SNPs within ROH in the Siberian Husky pet, show, sled, sled—sprint, and sled—distance populations was used to identify selective sweeps within these populations (Figure 5). ROH islands represent genomic regions where more than 50% of individuals in the population share overlapping ROH segments. A total of 91 ROH islands spanned 25 chromosomes (Supplementary Table S2). Show dogs had 52 ROH islands, more than twice as many as any other group, with an average island length of 1,510,879 bp. Pet and sled—distance dogs had similar numbers and lengths of ROH islands with 20 and 16 islands, respectively, and average lengths between 1.36 and 1.39 million base pairs. Sled dogs in total and sled—sprint dogs had the least ROH islands by far, with only 1 or 2 islands, all on CFA 5, and averaging only 325–370 thousand base pairs in length, respectively.

**Figure 6:**
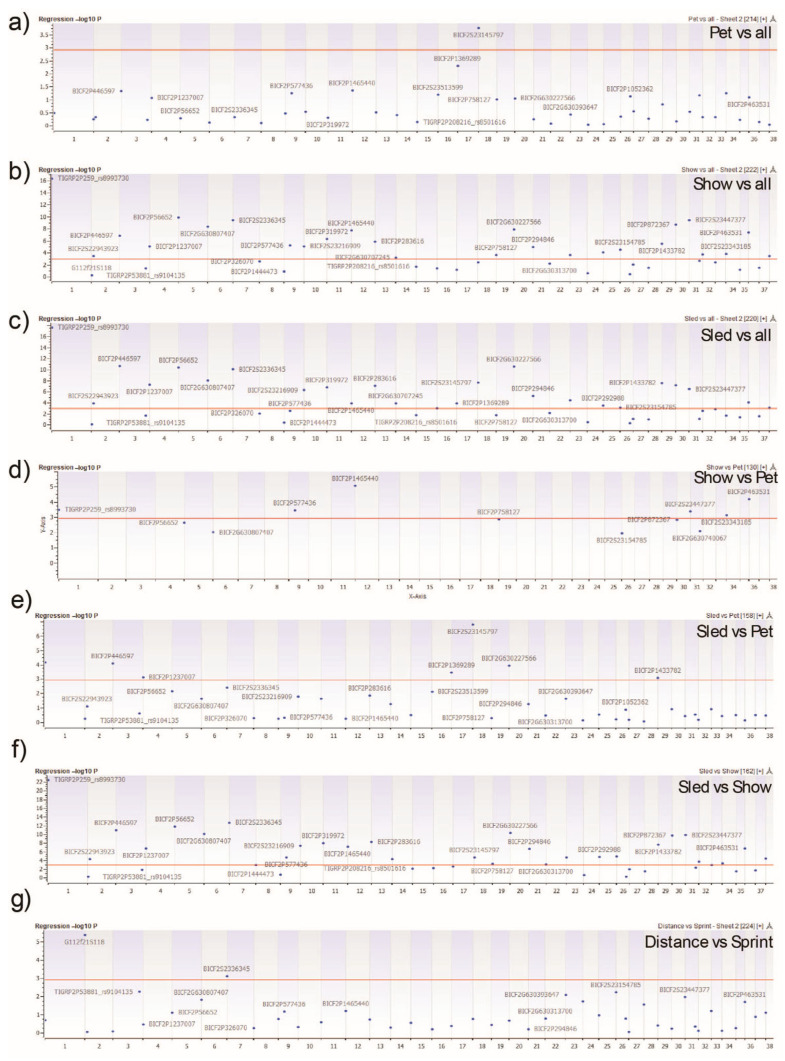
Homozygosity Association Tests Identify Runs of Homozygosity (ROH) Associated with Siberian Husky Populations. ROH clusters are represented by the leading SNP of each cluster and used in an association test comparing Siberian Husky populations. The panels (**a**) pet, (**b**) show, and (**c**) sled reflect a population as compared to all other Siberian Huskies. The remaining panels depict pairwise comparison between two individual populations of (**d**) show vs. pet, (**e**) sled vs. pet, (**f**) sled vs. show, and (**g**) distance vs. sprint. Genome location is displayed on the *x*-axis and degree of association is depicted on the *y*-axis. The horizontal red line represents the Bonferroni multiple testing correction threshold of a *p*-value ≤ 0.05.

**Figure 7:**
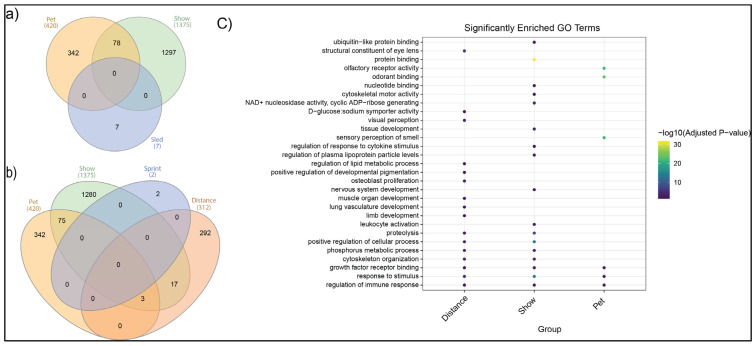
Overlap of genes within ROH islands and significant F_ST_ markers across populations and GO enrichment analysis. (**a**) A Venn diagram illustrating the number of shared and unique genes found within ROH and nearby F_ST_ markers among the Siberian Husky pet, show, and sled populations. (**b**) A Venn diagram illustrating the number of shared and unique genes found within ROH and nearby F_ST_ markers among the Siberian Husky pet, show, sled—distance, and sled—sprint populations. (**c**) A dot plot of significantly enriched Gene Ontology (GO) terms for genes found within ROH islands and genes annotated closest to significant F_ST_ markers. The color and size of each dot indicate the statistical significance of the enrichment (adjusted *p*-value).

The genome-wide homozygosity association tests allowed for the identification of ROH significantly differentiating Siberian Husky populations. Forty-two consensus ROH, referred to as ROH clusters, were identified in Siberian Huskies (Supplemental Table S3). These consensus ROH were present in all Siberian Husky populations. The average length of the ROH clusters was 50,838,107 bp (min 4,042,783 bp; max 122,421,400 bp). As with F_ST_, pet, show, and sled dogs were individually compared to all remaining Siberian Huskies, followed by subsequent pairwise comparisons of each population to one another and the sled subpopulations to one another, generating a total of seven genome-wide homozygosity association tests (Figure 6). All tests produced significant results, passing the Bonferroni multiple testing correction of a *p*-value less than 0.05. The pet versus all Siberian Huskies test produced a single ROH cluster found most commonly in pet dogs on CFA 18 (Figure 6a). The show dogs versus all Siberians (Figure 6b) and sled versus all Siberians (Figure 6c) tests identified the most ROH clusters (Show = 26; Sled = 24) significantly associated with their populations across the multiple tests. When conducting the one-on-one comparisons of pet-to-show and pet-to-sled (Figure 6d,e), 6 and 7 clusters, respectively, were significantly differentiated. Twenty-seven ROH clusters significantly differentiated the show-to-sled comparison (Figure 6f). Only two ROH clusters were significantly differentiated between the sled—sprint and —distance populations on CFA 2 and 7 (Figure 6g).

### 3.5. Gene Ontology and Pathway Enrichment Analysis

Gene ontology and pathway enrichment analysis were performed to understand the potential biological implications of the signatures of selection identified through marker-based F_ST_, ROH islands representing selective sweeps, and ROH association testing. Significant GO terms (FDR < 0.01) were identified separately for each analysis and redundant terms were summarized using REVIGO [27]. Only those terms and pathways that were enriched in all three analyses were considered significant. Supplemental Table S1 lists the nearest gene to each of the SNPs with an F_ST_ ≥ 0.6. Supplemental Table S2 lists the number of genes within the ROH islands identified and denotes those most likely to be biologically relevant. Supplemental Table S3 identifies the number of genes present in each ROH cluster in the association tests and any pathway enriched based upon the extensive number of genes in each of the clusters. To summarize these results, Venn diagrams highlight the overlap of genes identified in each Siberian Husky population (Figure 7a,b). There was overlap of genes only in the pet and show regions (*n* = 78) (Figure 7a) comparing the main purpose groups. However, when dividing the sled population into distance and sprint, additional gene overlap was found between sled—distance, show, and pet dogs (Figure 7b). Biological pathways significantly associated with the Siberian Husky pet, show, and sled—distance groups, with a measure of their degree of association, are shown in Figure 7c and Supplemental Table S4. Due to both sled and sled—sprint having very few ROH regions with genes, these populations were not assessed for pathway enrichment. Figure 7c utilizes gene lists identified by both F_ST_ and both ROH analyses for the pet, show, and sled—distance groups. Pathway enrichment analysis uniquely emphasized an overrepresentation of genes associated with sense of smell (olfactory receptor activity, odorant binding, and sensory perception of smell) in the pet dogs. Show dogs had an overrepresentation of genes related to protein and nucleotide binding, immune function, tissue development, cytoskeletal motor activity, and nervous system development. Sled dogs had an overrepresentation of genes related to the structural development of the eye lens and visual perception, as well as genes related to the development of muscle organs, lung vasculature, limbs, bones, and pigmentation. Additional genes selected for sled dogs were implicated in lipid metabolism and glucose transport.

## 4. Discussion

The Siberian Husky was originally developed in Alaska in the early 1900s as an Arctic sled dog breed of renowned speed and endurance. Over the past century, selection has diverged within the breed to focus on characteristics most desired for dogs who are still used traditionally as sled dogs versus those used in registered show competitions or as companion dogs. Prior research has identified genetic population structure in multiple dog breeds reflecting geographic isolation, differential breeding strategies, or strong selection for champion dogs [28]. Selection for working or sporting lineages as compared to show lineages has been noted as a main driver of within-breed population structure among breeds such as the Labrador Retriever (working versus show lines) and English Greyhound (sport versus show) [28]. With this said, not all dog breeds show a within-breed population structure, as demonstrated in a study of Nordic hunting breeds including the Finnish Spitz, Nordic Spitz, and the Karelian Bear Dog [29]. This study focused on the Siberian Husky breed for which a genomic population structure aligning with breeding purposes for sledding, show, and pet dogs has been seen with admixture and PCA results in our previous study of the Arctic sled dog breed history [3]. Here, our goal was to confirm the population structure, identify where in the genome selection is being imposed due to breeding purpose, and what the resulting biological implications are.

Our genomic PCA (Figure 1) replicated our previous findings with PC1 differentiating sled from show lineages. Both the PCA and F_ST_ results indicate more similarity between pet and show lineages with some pet dogs overlapping with show dogs in the PCA and no SNPs passing our F_ST_ threshold of 0.6, showing little differentiation between show and pet dogs. PC2 described further subpopulation divergence within the sledding group itself, separating the sprint from distance sledding dogs who are selected for speed or extreme endurance, respectively. This study also included owner-identified “dual-purpose” dogs; show–sled and pet–sled. Owners typically denoted the primary breeding priority as that noted first in our title description of show or pet dogs, respectively, and dogs commonly aligned with expectations and were merged with those groups for further analysis. However, dogs from two show–sled kennels uniquely grouped better with sled—sprint as opposed to show–sled. These dogs were grouped with sled—sprint for analysis and highlighted a greater degree of selection variation among the show–sled dogs.

In addition to our prior research identifying population structure within the Siberian Husky breed, personal observations of Siberian Husky morphology suggested conformational differences between show and sled dogs. Therefore, 18 morphometric measures were also collected on dogs to better understand selection differentiation among Siberian Husky purpose groups. Both ANOVA analyses (Table 1) and morphometric PCA (Figure 2) revealed body structure variation differentiating the pet, show, and sled groups. Some of the notable findings were that show dogs were generally smaller across most morphological measurements, whereas the sled population had an average wither height above the breed standard, with sled dog wither height averaging 60.22 cm (SD ± 3.43 cm) as compared to the AKC breed standard maximum height of 59.69 cm for males and 55.88 cm for females. PC2, representing a complex relationship between girth, foot circumferences, and leg lengths, clearly segregated pet, then show, then sled dogs. The only individual measurement to also distinguish the three groups but in a different order pattern of pet, then sled, then show, was body length. Pet dogs were the longest whereas the show dogs were the shortest in back length. PC3 and PC4 went on to differentiate the show or pet dog populations from the other purpose groups, respectively.

F_ST_, reflecting variation in allelic frequencies, and ROH, mapping selection for homozygosity, were used to identify regions of selective pressure within Siberian Husky usage groups. Show and sled populations were by far the two groups demonstrating the most allelic variation from one another, with 52 SNPs with an F_ST_ score greater than 0.6. Genes of biological interest in closest proximity to these F_ST_ markers included *IRF6*, *RUNX1*, and *DACT1*, all implicated in craniofacial or skeletal development, thereby supporting the genetic mechanisms potentially influencing the differences in body morphology between show and sled Siberian Huskies [30,31,32]. *FGGY*, *LDAH*, *PAPSS1*, and *SLC9A9* genes, associated with carbohydrate and lipid metabolism, may reflect less selective pressure in show dogs for athletic performance nowadays as compared to the sledding dogs for which athletic performance remains a top priority [33,34]. *SLC9A9* has also been associated with neurological function due to its high expression in the brain and association with neuropsychiatric disorders [35]. Sprint and distance sled dogs also produced a relatively high degree of allelic differentiation with 31 SNPs passing our threshold. Multiple genes including *HK2*, *ANXA1*, *KCNMA1*, and *SLC9A9* play roles in endurance and stamina by influencing metabolism and muscle function [35,36,37,38,39].

The identification of ROH highlights selection signatures within the genome and provides a measure of inbreeding when inherited from a common ancestor. Show dogs had the highest degree of inbreeding at 30.7%, 10–15% higher than pet and sled dog sub-populations. This reflects similar findings in our previous study of Siberian Husky populations and Arctic dog evolution [3]. Notably, in our previous study, 50% of sledding Siberian Huskies were found to be admixed with Alaskan sled dogs. Our current study did not investigate potential admixture and relied on AKC registration requiring three generations of registered ancestors as confirmation of breed purity, yet the admixture identified in the previous study also included breed registration as a prerequisite. Therefore, it is possible that admixture may exist and contribute to the decreased inbreeding levels in the sled population, particularly among the sled—sprint group, which had the lowest degree of inbreeding. In all, our inbreeding estimates reflect the greater number of ROH and longer length ROH present in show dogs, suggesting more recent inbreeding as compared to the primarily short length ROH in sled dogs (Figure 4), which reflects more historical inbreeding.

By calculating the frequency in which an SNP occurred in an ROH, selective sweeps were identified in each population, with those occurring in more than 50% of the population were referred to as ROH islands and deemed significant (Figure 5). Show dogs had more than double the ROH islands (*n* = 52) than pet and sled—distance dogs, which possessed the next highest number of ROH islands. Similar to the pairwise comparison of Siberian Husky groups in the F_ST_ analysis, the homozygosity association tests distinguished the show and sled populations, with at most 27 ROH regions significantly differentiating the two populations. Broader comparison of the show group to all other Siberians and the sled group to all other Siberians were similar, with 26 and 24 ROH passing the Bonferroni multiple testing correction. Notable genes in pet ROH regions include *IGF1R* and *ALX4*, previously associated with body size in dogs and blue eyes in Siberian Huskies, respectively [40,41]. Two genes, *CHRM4* and *DHCR7*, have been associated with behavior. *CHRM4* dysregulation can disrupt motor activities and lead to abnormal behavior, whereas *DHCR7* has been associated with enhanced sociability and tractability [35,42]. Again, supporting the variation in body size within Siberian Huskies, *LCORL*, *ACAN*, *FBN1*, *DSE*, *AFDN*, and *RYR3* all play roles in body size, skeletal and craniofacial morphology, and structural integrity through muscle and tissue regulation [35,43,44,45,46,47]. Distance sled dog ROH included genes such as *SMTN* and *SLC5A1*, both associated with smooth muscle function and glucose metabolism, respectively [34,35].

All genes highlighted by F_ST_ and ROH were assessed for biological pathway enrichment (Figure 7c). Multiple pathways were found to be significantly associated with gene lists related to the specific Siberian Husky purpose groups of pet, show, and sled dogs. Somewhat unexpectedly, pet dogs had an overrepresentation of genes associated with their sense of smell. Behavior attributes have also been linked to scenting ability [48]. Show dogs demonstrated selection related to protein and nucleotide binding, immune function, tissue development, cytoskeletal motor activity, and nervous system development. An interesting but unanswered question in this study is whether the selection of immune function parameters is an unintentional product of inbreeding depression or a response to purging heritable diseases from the show population. Signatures of selection are often expected to be the product of positive, intentional artificial, or natural selection for an advantageous trait, yet ROH also reflect increased autozyosity, where the same chromosomal region is inherited from a common ancestor. Increased autozygosity leads to inbreeding depression through the accumulation of recessive deleterious alleles [49]. This study does not include health information on the study population and, as such, is unable to determine whether homozygous regions are selecting for an advantageous trait or the product of inbreeding. The sledding population was particularly enriched for genes related to development and metabolism. Specifically, the structural development of the eye lens and visual perception and the development of muscle organs, lung vasculature, limbs, bones, and pigmentation were enriched. One theory related to the enrichment for pigmentation might be due to less pressure on sledding Siberian Huskies to conform to the most popular breed coat color patterns seen in the show and pet populations.

## 5. Conclusions

This study was one of the first of its kind, where divergent selection within a dog breed was not only identified, but further assessed for biological significance. Signatures of selection do not provide confirmation of how genomic regions and the genes within are specifically impacted. Intentional selection, whether artificial or in response to a natural pressure, is often considered to be positive and to provide an advantage to the individual. However, selective sweeps and increased autozygosity due to inbreeding can also harbor deleterious mutations. As such, this study identifies genes and the dominating biological pathways under selection in the Siberian Husky breed due to varying prioritization of characteristics desired in pet, show, or sled dogs but does not determine impact outside of the direct morphological measures included in the study. Excitingly, many of the enriched pathways make plausible sense with expectations of why and how they are likely influenced due to the specific breeding purposes in the Siberian Husky. In all, this study provides the first evidence of the biological impact of genetic selection within the Siberian Husky breed for the distinct purposes of sledding, showing, and as pets, while simultaneously maintaining overall population uniformity to meet breed standards.

## Figures and Tables

**Table 1:** Summary of body measurements across groups. Values are presented as mean ± standard deviation for each trait.

Trait (Measured in cm)	Pet	Show	Sled
Eye Width *	4.45 ± 0.53 ^a^	4.42 ± 0.64 ^a^	4.11 ± 0.81 ^b^
Snout Length	9.45 ± 0.97 ^a^	9.19 ± 0.91 ^a^	9.42 ± 1.17 ^a^
Outside Ear Length	8.79 ± 1.30 ^a^	8.43 ± 0.89 ^a^	8.38 ± 1.22 ^a^
Chest Width *	13.94 ± 1.98 ^a^	12.29 ± 1.40 ^b^	12.65 ± 1.35 ^b^
Neck Girth	35.99 ± 3.81 ^a^	37.41 ± 3.86 ^a^	36.42 ± 3.81 ^a^
Chest Girth *	67.23 ± 6.78 ^a^	62.56 ± 4.55 ^b^	64.85 ± 4.47 ^ab^
Wither Height *	56.92 ± 3.84 ^a^	56.85 ± 3.66 ^a^	60.22 ± 3.43 ^b^
Height at Base of Tail *	55.32 ± 4.01 ^a^	54.03 ± 4.04 ^a^	58.37 ± 5.97 ^b^
Body Length *	61.85 ± 6.68 ^a^	54.69 ± 4.52 ^b^	57.68 ± 4.34 ^c^
Tail Length	35.71 ± 4.67 ^a^	35.84 ± 2.87 ^a^	34.42 ± 4.42 ^a^
Upper Foreleg Length *	20.04 ± 2.41 ^a^	19.10 ± 1.91 ^a^	17.83 ± 2.64 ^b^
Lower Foreleg Length	21.41 ± 2.03 ^a^	20.93 ± 1.55 ^a^	21.29 ± 1.83 ^a^
Fore Foot Length *	13.56 ± 1.60 ^a^	12.50 ± 1.55 ^b^	12.83 ± 1.50 ^ab^
Fore Foot Circumference	9.91 ± 0.86 ^a^	10.24 ± 1.24 ^a^	10.13 ± 0.86 ^a^
Upper Hind Leg Length *	30.51 ± 3.33 ^a^	27.30 ± 2.36 ^b^	30.15 ± 3.38 ^a^
Lower Hind Leg Length *	24.21 ± 2.92 ^a^	23.60 ± 2.24 ^a^	22.00 ± 3.20 ^b^
Hind Foot Length *	21.23 ± 2.01 ^a^	20.80 ± 1.60 ^ab^	20.24 ± 2.24 ^b^
Hind Foot Circumference	9.47 ± 0.86 ^a^	9.63 ± 1.04 ^a^	9.68 ± 0.86 ^a^

Traits marked with an asterisk (*) showed significant differences across groups based on one-way ANOVA (*p* < 0.05). Superscript letters indicate Tukey HSD groupings; groups sharing the same letter are not significantly different. All reported values are in centimeters.

## Data Availability

The genotypic data and breed information will be released in a public repository upon paper acceptance.

## Supplementary Materials

The following supporting information can be downloaded at: https://www.mdpi.com/article/10.3390/genes16111355/s1, File S1: Morphological measurements taken on dogs, Figure S1: Scatter plots of the top 4 principal components of the morphometric measurements; Tables S1–S4: Results on F_ST_ and ROH regions.

## Abbreviations

The following abbreviations are used in this manuscript:

AKC	American Kennel Club
ROH	Runs of homozygosity
SHCA	Siberian Husky Club of America
SNP	Single-nucleotide polymorphism
