# Supplementary material for: Breeding Selection for U.S. Siberian Huskies Has Altered Genes Regulating Metabolism, Endurance, Development, Body Conformation, Immune Function, and Behavior
Source: Genes (Basel). 2025 Nov 10;16(11):1355. doi: 10.3390/genes16111355 (PMC12652727; doi:10.3390/genes16111355)
Supplement: Supplementary file 1 [file genes-16-01355-s001.zip › File S1_Morphological measurement definitions.pdf]

Supplemental File S1

Morphological measurements taken on dogs

Measurements and definitions following Sutter, N.B., et al., *Morphometrics within dog breeds are highly reproducible and dispute Rensch's rule.* Mammalian Genome, 2008. **19**(10): p. 713-723.

Eye width – The linear distance between the punctae lacrimale of the left eye and the punctae lacrimale of the right eye.

Snout length – The distance along the skull from the rostral end of the planum nasale to dorsal plane between the punctae lacrimale of the left eye and the punctae lacrimale of the right eye (stop).

Outside ear length – The linear distance from the base of the outside of the ear (the divot where the ear meets the skull) straight up to the pointed tip of the ear.

Chest width – The linear distance along the dorsal plane from the greater tubercle of the left humerus to the greater tubercle of the right humerus.

Neck girth – The circumference of the neck at the median distance between the external occipital protuberance and the withers.

Chest girth – The circumference of the deepest part of the thoracic cavity in a plane with both the sternum and withers.

Wither height – The linear distance from the ground to the cranial angle of the scapula (withers). Measured when the dog is in an upright, stacked position.

Height at base of tail – The linear distance from the ground to the dorsal-most point where the tail meets the body. Measured when the dog is in an upright, stacked position.

Body length – The distance along the body from the dorsal plane of the withers to the cranial-most point where the tail meets the body. This is measured along the median plane of the dog.

Tail length – The linear distance from the base of the tail where the tail meets the body, to the tip of the tail where the vertebrae or bone ends. Do not include the extra hair at the tip of the tail.

Upper foreleg length – The linear distance from the olecranon process of the ulna to the greater tubercle of the humerus.

Lower foreleg length – The linear distance from the free epiphysis of the accessory carpal bone to the olecranon process of the ulna. This is measured along the lateral aspect of the dog.

Fore foot length - The distance along the ventral side of the fore foot from the distal end of the fourth digit, not including the claw, along the digital and metacarpal pads up to the free epiphysis of the accessory carpal bone.

Fore foot circumference - The circumference of the fore foot at the median distance between the free epiphysis of the accessory carpal bone to the metacarpal pad.

Upper hind leg length – The linear distance from the patella to the dorsal-most point where the tail meets the body.

Lower hindleg length – The linear distance from the hock to the patella. This is measured along the lateral aspect of the dog.

Hind foot length – The distance along the ventral side of the hind foot from the distal end of the fourth digit, not including the claw, along the digital and metatarsal pads up to the calcaneal tuberosity (hock).

Hind foot circumference - The circumference of the hind foot at the median distance between the hock and the metatarsal pad.
