# Supplementary figures and images for: Breeding Selection for U.S. Siberian Huskies Has Altered Genes Regulating Metabolism, Endurance, Development, Body Conformation, Immune Function, and Behavior
Source: Genes (Basel). 2025 Nov 10;16(11):1355. doi: 10.3390/genes16111355 (PMC12652727; doi:10.3390/genes16111355)

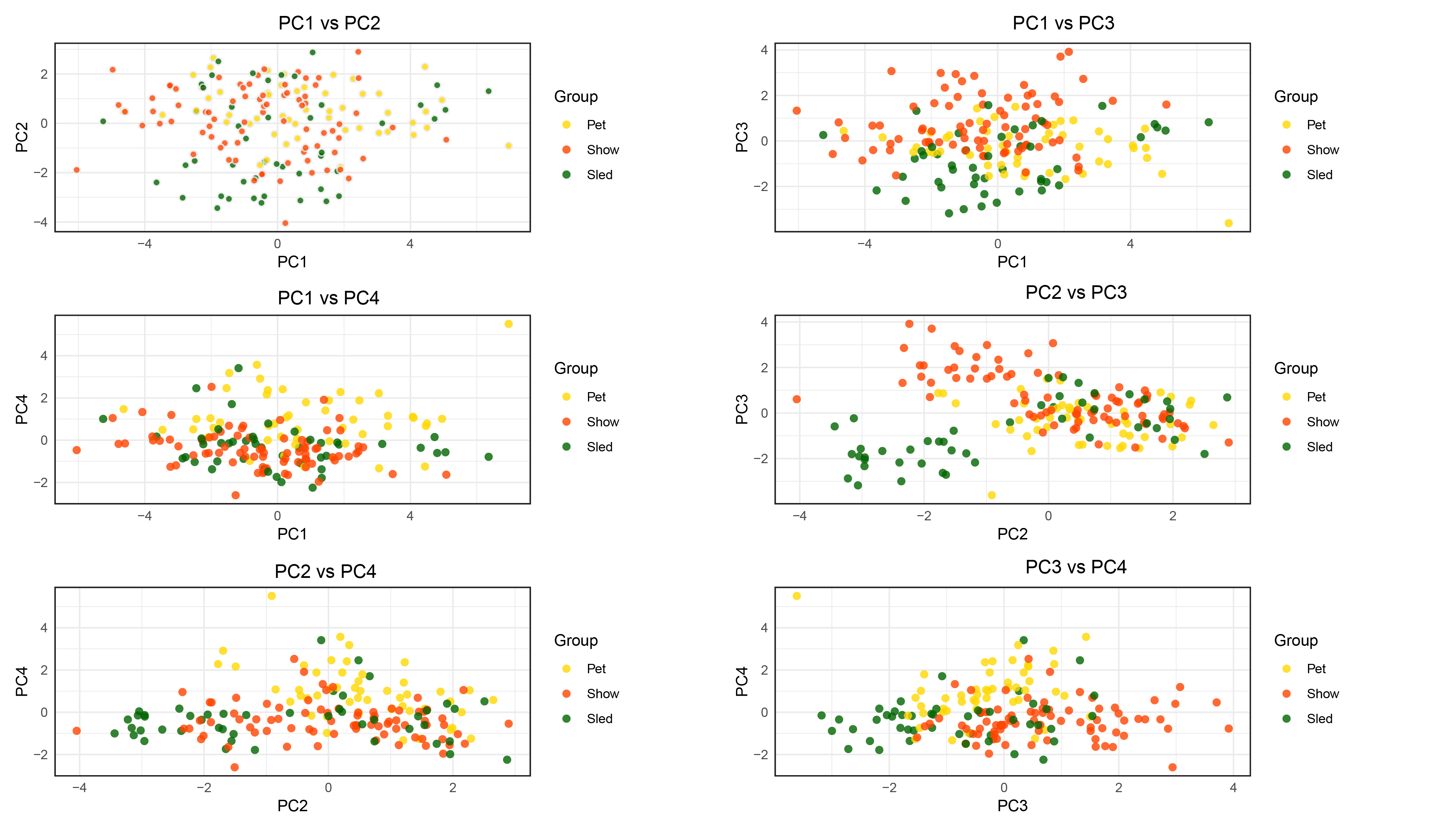

Supplement: Supplementary file 1 [file genes-16-01355-s001.zip › Figure S1_Multiple_PC_Morphometric.png]
